# Characteristics of chemokine signatures elicited by EGF and TNF in ovarian cancer cells

**DOI:** 10.1186/1476-9255-10-25

**Published:** 2013-06-25

**Authors:** Deok-Soo Son, Syeda M Kabir, Yuanlin Dong, Eunsook Lee, Samuel E Adunyah

**Affiliations:** 1 Department of Biochemistry and Cancer Biology, Meharry Medical College, Nashville, TN, USA; 2 Department of Physiology, Meharry Medical College, Nashville, TN, USA

**Keywords:** Chemokines, NF-κB, TNF, EGF, Ovarian cancer

## Abstract

**Background:**

Ovarian cancer, an inflammation-associated cancer, is the fifth leading cause of cancer deaths in women. The malignancy produces a large amount of tumor necrosis factor (TNF) which promotes a proinflammatory tumor microenvironment. In addition, the epidermal growth factor receptor (EGFR) is frequently overexpressed in high-grade ovarian cancer, which likely aggravates cancer progression. Since ovarian cancer progression is closely associated with chemokine networks driven by inflammation or EGFR activation, we investigated the chemokine signatures elicited by EGF and TNF in ovarian cancer cells to determine their individual profiles and if there was in fact some kind of synergy between their actions on the chemokine network.

**Methods:**

We used a PCR array for the chemokine network to examine the signature of chemokines and their receptors elicited by EGF and TNF in four ovarian cancer cell lines (OVCAR-3, SKOV-3, CaOV-3 and TOV-21G).

**Results:**

The chemokine network revealed that ovarian cancer cells commonly expressed high levels of proinflammatory chemokines such as CCL20, CXCL1-3 and CXCL8 in response to EGF or TNF. However, the responsiveness to EGF or TNF displayed a cell line specific pattern. Although OVCAR-3 and SKOV-3 cells were responsive to either EGF or TNF, their TNF responsiveness was dominant. On the other hand, CaOV-3 and TOV-21G cells were responsive to EGF but less to TNF, probably due to the high levels of non-canonical nuclear factor (NF)-κB components such as IKKα and p52 in these cell lines compared to OVCAR-3 and SKOV-3 cells. Among chemokine receptors, only CXCR5 was responsive to EGF or TNF in CaOV-3 cells. Finally, CCL20 and CXCL8 responded synergistically in response to EGF and TNF in OVCAR-3 and SKOV-3 cells.

**Conclusion:**

Our results indicate that CCL20, CXCL1-3 and CXCL8 are the primary chemokines induced by EGF or TNF and are elicited in these ovarian cancer cells via NF-κB, Akt and Erk signaling pathways. Of interest, there was a syngergistic response in terms of CCL20 and CXCL8 levels, when OVCAR-3 and SKOV-3 cells were exposed to EGF plus TNF. Targeting these proinflammatory chemokines may be a promising therapeutic strategy for ovarian cancer with abundant TNF and EGFR activation patterns.

## Background

Inflammation-associated ovarian cancer is the fifth leading cause of cancer deaths among women. Chronic infection and inflammation are considered to be among the most important epigenetic and environmental factors contributing to tumorigenesis and cancer progression [1-3]. Ovarian cancer cells highly express highly tumor necrosis factor (TNF), indicating the importance of TNF as a regulator of the proinflammatory tumor microenvironment in this malignancy [3-6]. Thus blockage of the TNF network using neutralizing antibodies or siRNA reduces the ovarian cancer burden [7]. Particularly, TNF regulates chemokine networks through the nuclear factor-κB (NF-κB) signaling pathway. Chemokines are critical mediators of cell migration into and out of the tumor microenvironment and represent major players in cancer progression and metastasis [8,9]. In our previous study, inflammatory agents including bacterial endotoxin lipopolysaccharide (LPS) and proinflammatory cytokines interleukin-1 (IL-1) and TNF, induced CXCL1-3 and CXCL8 in ovarian cancer cells by involving NF-κB signaling [10]. In addition, those cells with a high endogenous expression of TNF expressed higher levels of CXCR4 than cells with a low TNF expression level [11].

The ErbB/EGFR family of receptors consists of four structurally-related type 1 transmembrane tyrosine kinase receptors: EGFR (ErbB1, HER1), ErbB2 (HER2, neu), ErbB3 (HER3) and ErbB4 (HER4) [12,13]. Activation of ErbB/EGFR tyrosine kinase receptors recruits multiple signaling pathways which play important roles in cell proliferation, survival, adhesion, motility, invasion and angiogenesis [12,13]. EGFR is frequently overexpressed in high-grade malignant ovarian cancer [14,15]. Because of the multiple signaling processes originating from EGFR activation, EGFR overexpression has been correlated with a poor prognosis and a decreased therapeutic responsiveness in patients with ovarian cancer [12]. The phosphatidylinositol 3-kinase (PI3K) which acts as a main downstream signaling pathway for EGF is frequently activated, leading to increased cell survival. Furthermore, EGFR activation may also involve chemokine networks through its downstream signaling pathways. CXCL1 and CXCL8, in particular, have been identified as secreted proteins regulated by EGF and the PI3K pathway in ovarian cancer cell lines [16]. In addition, EGF enhances the expression of CXCR4 as well as migration of ovarian cancer cells [17].

These limited reports indicate that ovarian cancer progression is closely associated with chemokine networks driven by inflammation or EGFR activation. Therefore it is important to identify which chemokines are primarily regulated by TNF and EGF to order to devise a therapeutic strategy based on the chemokine signature seen in this malignancy. Thus the present study was designed to assess the characteristics of the chemokine profiles elicited by EGF and TNF, and determine if they induce any synergistic effects with respect to key chemokines in ovarian cancer cell lines. For the purposes of this study, we evaluated four such lines: OVAR-3, SKOV-3, CaOV-3 and TOV-21G.

## Methods

### Reagents

Recombinant human TNF and EGF were obtained from R&D Systems (Minneapolis, MN, USA). Antibodies were purchased from the following vendors: ErbB isoform, p65 and β-actin from Santa Cruz Biotechnology (Santa Cruz, CA, USA) and NF-κB family, IKK isoforms, IκB, Akt, Erk and their phosphorylated forms from Cell Signaling Technology (Beverly, MA, USA). The PCR array for customized human chemokines, specific PCR primers for CCL2, CCL20, CXCL1-3, CXCL8, CXCL16, CXCR5, β-actin, and SYBR® Green Master Mix came from SABiosciences/Qiagen (Frederick, MD, USA). Chemiluminescent detection kits were from GE Healthcare (Piscataway, NJ, USA). Penicillin G/streptomycin was purchased from Sigma (St. Louis, MO, USA) and Lipofectamine 2000 and all liquid culture media were acquired from Invitrogen (Grand Island, NY, USA). BAY-11-7082, LY294002 and PD98059 were purchased from Cayman Chemical (Ann Arbor, MI, USA). The Luciferase Reporter Assay System was obtained from Promega (Madison, WI, USA).

### Cell lines, cell culture, and media additions

The human ovarian cancer cell lines OVCAR-3, SKOV-3, CaOV-3 and TOV-21G were purchased from the American Type Culture Collection (ATTC, Manassas, VA, USA). Human cancer cells (approximately 5 × 10^4^ cells/ml) were cultured at 37°C in a water-saturated atmosphere of 95% air and 5% CO^2^ in 24- or 6-well plates with RPMI medium containing 10% fetal bovine serum (FBS) with penicillin (100 U/ml)/streptomycin (100 U/ml). After an overnight culture to allow cellular attachment to the plates, the medium was removed and fresh medium without FBS was added to remove the effects of serum, per se. Where indicated, vehicle (phosphate-buffered saline, PBS), 10 ng/ml EGF, 10 ng/ml TNF, or a combination of EGF and TNF was added, and incubations continued for the indicated time periods.

### PCR array and real-time PCR

After isolating total RNA and eliminating genomic DNA, reverse transcription reactions were performed at 42°C for 15 min followed by 94°C for 5 min. According to manufacturers’ instructions, a real-time PCR was performed using a Bio-Rad CFX96 (Hercules, CA, USA) under the following two-step cycling program: 1 cycle at 95°C for 10 min, 40 cycles at 95°C for 15 sec and at 60°C for 1 min. Data analysis was performed based on a Web-Based PCR Array Data Analysis protocol (http://pcrdataanalysis.sabiosciences.com/pcr/arrayanalysis.php) provided by SABiosciences (Qiagen).

### Western blots

Cell lysates were prepared, resolved on SDS-polyacrylamide gels and transferred to nitrocellulose membranes according to established procedures [13]. Blocking of nonspecific proteins was performed by incubation of the membranes with 5% nonfat dry milk in Tris buffered saline Tween-20 (TBST, pH 8.0) for 2 h at room temperature. Blots were incubated with primary antibodies at 1:1,000 dilution in blocking solution overnight at 4°C. The membranes were washed 3 times with TBST for 10 min and incubated for 1 h with horseradish peroxidase-conjugated secondary antibody at 1:2,500 in 5% milk/TBST. The membranes were then rinsed 3 times with TBST for 10 min and the bands were visualized by enhanced chemiluminescence. After membrane stripping for 10 min with methanol containing 3% H_2_O_2_, β-actin was detected in order to serve as an internal loading control.

### Construction of the CCL20 and CXCL8 promoter-luciferase genes

DNA fragments of the human CCL20 and CXCL8 genes were generated by PCR using genomic DNA isolated from OVCAR-3 cells. Primer sets were designed as follows: 5′-GGA GTT CTG GAA TGT TCC TG -3′ for a sense containing a *XhoI* site and 5′-TAC CCA GTT CTT TGG GAG TG-3′ for an antisense containing a *HindIII* site for the CCL20 promoter (−376/+20) and 5′-CAC CTG CCA CTC TAG TAC TA-3′ for a sense containing a *XhoI* site and 5′-CCT TAT GGA GTG CTC CGG TG-3′ for an antisense site containing a *HindIII* site for the CXCL8 promoter (−322/+10). The PCR reaction was performed for 35 cycles at 94°C for 30 sec, 58°C for 30 sec and 74°C for 1 min with a final extension at 74°C for 10 min. The amplified CCL20 and CXCL8 DNA fragments were digested with *XhoI* and *Hind III* and the fragments were purified according to manufacturer‘s instructions (Gel Extraction System, Qiagen, Valencia, CA). The purified CCL20 and CXCL8 promoter genes were subcloned into the *XhoI* and *HindIII* sites of the pGL4.12-basic vector (Promega, Madison, WI, USA). The constructs of the CCL20 and CXCL8 promoter-luciferase genes were confirmed by DNA sequencing analysis.

### Transient transfection and luciferase assays

Human ovarian cancer cells at approximately 50% confluency in 24-well plates were washed once with fresh media without additives and were transiently transfected for 24 h at 37°C using Lipofectamine solution. Transfected cells were treated as outlined in Results and incubated for 6 h. After rinsing cells with ice-cold PBS and adding lysis buffer (Promega, Madison, WI), cell lysates were used for determination of luciferase activity using a microplate luminometer. Luciferase activity, expressed as relative light units, was normalized to measured protein levels.

### Statistical analysis

Data were analyzed by the paired Student’s *t*-test and one-way analysis of variance (ANOVA) as appropriate. If a statistical significance (P ≤ 0.05) was determined by ANOVA, the data were further analyzed by Tukey’s pairwise comparisons to detect specific differences between treatments.

## Results

### EGF- or TNF-responsive chemokine signature in ovarian cancer cells

We selected ovarian cancer cell lines OVCAR-3, SKOV-3, CaOV-3 and TOV-21G to determine PCR arrays containing genes that encode human chemokines and chemokine receptors after the addition of EGF or TNF. The present study used the nomenclature of chemokines approved by the IUIS/WHO Subcommittee on Chemokine Nomenclature (2002). The mRNA levels of a panel of 43 chemokines and 19 chemokine receptors were evaluated for each of the 4 cell lines. Based on a Web-Based PCR Array Data Analysis protocol provided by SABiosciences (Qiagen), the absent, low and high expression levels of chemokines were defined as >35, 30–35 and <30 average threshold cycles, respectively.

**Figure 1:**
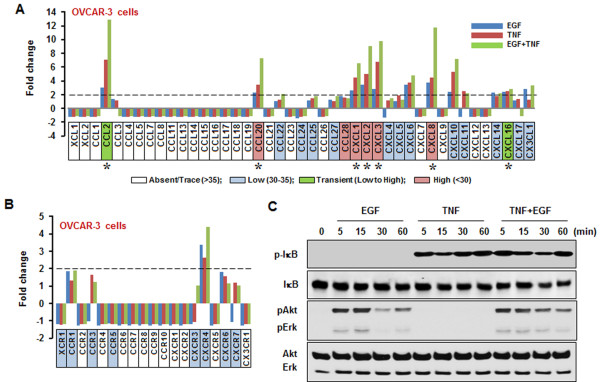
**EGF- or TNF-responsive chemokine signatures in OVCAR-3 cells.** EGF- or TNF-responsive chemokines (**A**) and chemokine receptors (**B**) were determined by PCR array. Cells were incubated with vehicle (Control), EGF (10 ng/ml), TNF (10 ng/ml) or both for 1 h. Values shown are fold changes compared to vehicle controls. After isolating total RNA, a real-time PCR was performed using a customized human chemokine PCR array. The dashed lines in (**A**) and (**B**) indicate the equivalent of 2-fold changes from controls. A chemokine signature with a >2-fold increase was considered to be EGF- or TNF-responsive. White, blue, green and red blocks indicate absent, low, transient (low to high) and high expression levels of chemokines, respectively, based on a >35, 30–35, 30–35 to <30, and <30, on average, threshold cycle. An asterisk (*) indicates EGF- or TNF-responsive chemokines with a high expression level (below an average threshold cycle of 30). (**C**) Effects of EGF or TNF on IκB, Akt and Erk activation. OVCAR-3 cells were treated with EGF (10 ng/ml) or TNF (10 ng/ml), or both, for 0–60 min. Whole cell lysates were prepared and IκB, Akt and Erk activations were confirmed by Western blot analyses. Experiments were performed in duplicate and a representative result is shown.

OVCAR-3 cells highly expressed CCL20, CCL28, CXCL1, CXCL2, CXCL3 and CXCL8. Except for CCL28, all were responsive to EGF or TNF (Figure 1A and Table 1). Although control CCL2 and CXCL16 cytokines were expressed at low levels, they were both highly elevated following the addition of EGF or TNF. The effects on CCL2, CCL20 and CXCL8 levels appeared to be synergistic when both EGF and TNF were added (Figure 1A and Table 1). Although CXCL6, CXCL10 and CX3CL1 were induced by EGF or TNF, overall they had a low expression (Figure 1A). OVCAR-3 cells displayed absent or low chemokine receptor levels. Although CXCR4 was responsive to EGF or TNF, the expression levels post addition of either factor, were still low (Figure 1B). In addition, EGF activated Akt and Erk whereas TNF solely activated IκB in OVCAR-3 cells (Figure 1C).

**Table 1:** EGF- and/or TNF-responsive chemokines and chemokine receptors obtained in ovarian cancer cell lines

	**OVCAR-3**			**SKOV-3**			**CaOV-3**			**TOV-21G**		
**Chemokine**	**EGF**	**TNF**	**ET**	**EGF**	**TNF**	**ET**	**EGF**	**TNF**	**ET**	**EGF**	**TNF**	**ET**
**CCL2**	**2.95**	**7.01**	**12.86**							1.28	-1.03	1.38
**CCL20**	**2.20**	**3.41**	**7.19**	**3.68**	**2.96**	**23.26**	**2.66**	1.20	**3.10**			
**CCL25**							1.02	1.37	1.43	1.31	1.39	1.17
**CCL26**				-1.44	-1.11	1.02						
**CCL28**	1.77	1.55	1.42	-1.06	-1.02	-1.08	-1.09	1.21	1.06	-1.11	1.21	-1.25
**CXCL1**	**2.53**	**4.38**	**6.48**	**2.64**	**8.03**	**10.56**	1.72	1.59	**2.25**	**3.02**	1.07	**3.23**
**CXCL2**	**3.43**	**4.99**	**8.97**	1.87	**13.98**	**12.82**	**5.82**	1.83	**7.67**	**4.81**	1.04	**5.82**
**CXCL3**	**2.81**	**6.73**	**9.68**	1.45	**9.09**	**10.85**	1.30	1.55	1.82	**2.80**	1.25	**3.51**
**CXCL5**										1.28	-1.00	1.33
**CXCL8**	**3.73**	**4.38**	**11.67**	**6.19**	**9.75**	**35.02**	**5.74**	1.90	**9.85**	**5.15**	1.00	**5.74**
**CXCL12**										-1.33	1.26	-1.13
**CXCL14**							-1.24	1.19	-1.04	-1.14	1.23	-1.03
**CXCL16**	**2.40**	**2.50**	**2.74**	-1.02	1.00	-1.23	1.12	1.33	1.09	-1.04	1.20	-1.09
**Receptor**												
**CCR10**										-1.36	1.14	-1.25
**CXCR4**							1.32	1.70	1.62	1.38	1.15	1.27
**CXCR5**							**5.24**	**3.33**	**10.63**			
**CXCR6**										1.08	1.24	-1.06
**CXCR7**							-1.10	-1.04	-1.44			

Only PCR array data for highly expressed chemokines and chemokine receptors (below an average threshold cycle of 30) are described above. Values shown represent the fold changes over baseline values, in the presence of EGF, TNF or both (ET). Bold numbers indicate EGF- or TNF-responsive chemokines and chemokine receptors levels that were elevated > 2-fold among highly expressed chemokines. Bold underlined numbers indicate the presence of a significant synergism when both EGF and TNF were added.

**Figure 2:**
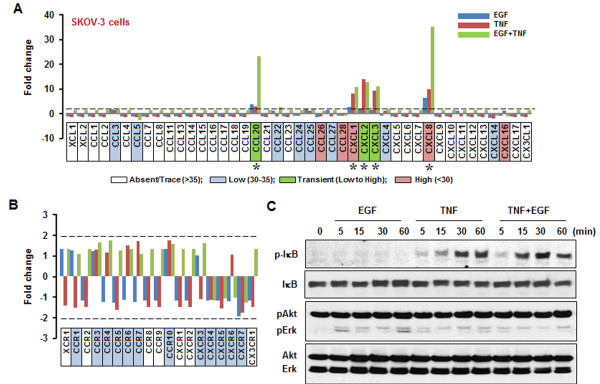
**EGF- or TNF-responsive chemokine signatures in SKOV-3 cells.** EGF- or TNF-responsive chemokines (**A**) and chemokine receptors (**B**) were determined by PCR array. Cells were incubated with vehicle (Control), EGF (10 ng/ml), TNF (10 ng/ml) or both for 1 h. Values shown are fold changes compared to vehicle controls. After isolating total RNA, a real-time PCR was performed using a customized human chemokine PCR array. Dashed lines in (**A**) and (**B**) indicate the equivalent of 2-fold changes from controls. A chemokine signature with a >2-fold increase was considered to be EGF- or TNF-responsive. White, blue, green and red blocks indicate absent, low, transient (low to high) and high expression levels of chemokines, respectively, based on a >35, 30–35, 30–35 to <30, and <30, on average, threshold cycle. An asterisk (*) indicates EGF- or TNF-responsive chemokines with a high expression level (below an average threshold cycle of 30). (**C**) Effects of EGF or TNF on IκB, Akt and Erk activation. Cells were treated with EGF (10 ng/ml) or TNF (10 ng/ml) to both for 0–60 min. Whole cell lysates were prepared and IκB, Akt and Erk activations were confirmed by Western blot analyses. Experiments were performed in duplicate and a representative result is shown.

SKOV-3 cells expressed high levels of CCL26, CCL28, CXCL1, CXCL8 and CXCL16; of these, CXCL1 and CXCL8 were responsive to either EGF or TNF (Figure 2A and Table 1). Although CCL20, CXCL2 and CXCL3 were expressed at low levels in the absence of EGF or TNF, they were highly expressed by EGF and TNF. Based on the fact that the level of CCL20 and CXCL8 in SKOV-3 cells exposed to both EGF and TNF were greater than the sum of the levels reached with either EGF or TNF alone, we judged that in these cases, there was a synergistic effect of EGF and TGF in these cells (Figure 2A and Table 1). In SKOV-3 cells chemokine receptor levels were either absent or low and had no responsiveness to either EGF or TNF (Figure 2B). SKOV-3 cells had a constitutively high level of phosphorylated (activated) Akt that did not appear to be influenced by the addition of EGF or TNF. However EGF activated Erk without IκB phosphorylation whereas TNF highly activated IκB and had a small effect on the activation of Erk (Figure 2C).

**Figure 3:**
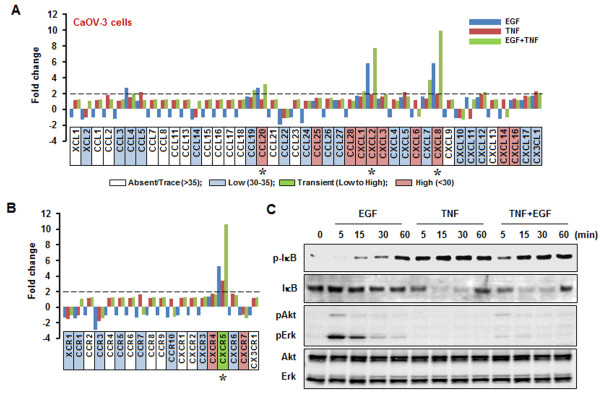
**EGF- or TNF-responsive chemokine signatures in CaOV-3 cells.** EGF- or TNF-responsive chemokines (**A**) and chemokine receptors (**B**) were determined by PCR array. Cells were incubated with vehicle (Control), EGF (10 ng/ml), TNF (10 ng/ml) or both for 1 h. Values shown are fold changes compared to vehicle controls. After isolating total RNA, a real-time PCR was performed using a customized human chemokine PCR array. Dashed lines indicate the equivalent of 2-fold changes from controls. A chemokine signature with a >2-fold increase was considered as EGF- or TNF-responsive. White, blue, green and red blocks indicate an absent, low, transient (low to high) and high expression level of chemokines, respectively, based on a >35, 30–35, 30–35 to <30 and <30, on average, threshold cycle. An asterisk (*) indicates EGF- or TNF-responsive chemokines with a high expression level (below an average threshold cycle of 30). (**C**) Effects of EGF or TNF (or both) on IκB, Akt and Erk activation. Cells were treated with EGF (10 ng/ml) or TNF (10 ng/ml) for 0–60 min. Whole cell lysates were prepared and IκB, Akt and Erk activations were confirmed by Western blot analyses. Experiments were performed in duplicate and a representative result is shown.

CaOV-3 cells highly expressed CCL20, CCL25, CCL28, CXCL1, CXCL2, CXCL3, CXCL6, CXCL8, CXCL14 and CXCL16, of which CCL20, CXCL2 and CXCL8 were more responsive to EGF than TNF (Figure 3A and Table 1). CXCL8 appeared to be synergistically elevated in the presence of both EGF and TNF (Figure 3A and Table 1). CaOV-3 cells displayed high basal levels of CXCR4 and CXCR7 that were not responsive to either EGF or TNF. Although CXCR5 level was low, it was highly expressed in response to EGF or TNF (Figure 3B and Table 1). EGF activated IκB, Akt and Erk whereas TNF activated only IκB and reduced EGF-activated Akt and Erk in these cells (Figure 3C).

**Figure 4:**
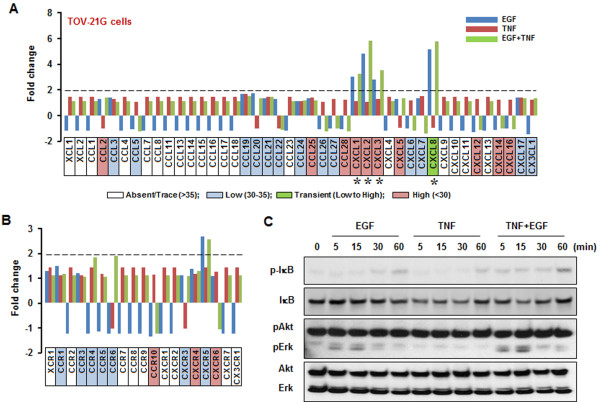
**EGF- or TNF-responsive chemokine signatures in TOV-21G cells.** EGF- or TNF-responsive chemokines (**A**) and chemokine receptors (**B**) were determined by PCR array. Cells were incubated with vehicle (Control), EGF (10 ng/ml), TNF (10 ng/ml) or both for 1 hour. Values shown are fold changes compared to vehicle controls. After isolating total RNA, a real-time PCR was performed using a customized human chemokine PCR array. Dotted lines indicate the equivalent of 2-fold changes from controls; chemokine signature with a greater than 2-fold increase is recognized as EGF- or TNF-responsiveness. White, blue, green and red blocks indicate absent, low, transient (low to high) and high expression levels of chemokines, respectively, based on a >35, 30–35, 30–35 to <30 and <30, on average, threshold cycle. Asterisk indicates EGF- or TNF-responsive chemokines with a high expression level (below an average threshold cycle of 30). (**C**) Effects of EGF or TNF on IκB, Akt and Erk activation. Cells were treated with EGF (10 ng/ml) or TNF (10 ng/ml) for 0 to 60 min. Whole cell lysates were prepared and IκB, Akt and Erk activations were confirmed by Western blot analyses. Experiments were performed in duplicate and a representative result is shown.

Lastly, TOV-21G cells highly expressed CCL2, CCL25, CCL28, CXCL1, CXCL2, CXCL3, CXCL5, CXCL12, CXCL14 and CXCL16, of which CXCL1, CXCL2 and CXCL3 were more responsive to EGF than TNF (Figure 4A and Table 1). Although baseline TOV-21G cellular CXCL8 values were expressed at low levels, it was highly expressed by EGF (Figure 4A and Table 1). TOV-21G cells displayed high levels of CCR10, CXCR4 and CXCR6, which were unresponsive to EGF or TNF (Figure 4B and Table 1). Although CXCR5 was responsive to EGF or TNF, its expression level was still low (Figure 4B and Table 1). TOV-21G cells had constitutively high Akt activation levels. EGF activated IκB and Erk whereas TNF activated only IκB (Figure 4C).

### Confirmation of EGF- or TNF-responsive chemokines in ovarian cancer cells

**Figure 5:**
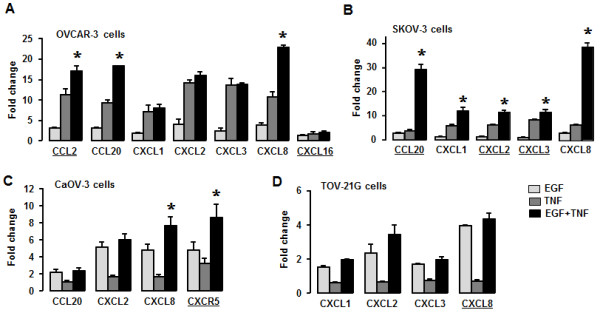
**Confirmation of EGF- or TNF-responsive chemokines in ovarian cancer cell lines.** (**A**) Effects of EGF or TNF on CCL2, CCL20, CXCL1, CXCL2, CXCL3, CXCL8 and CXCL16 by qRT-PCR in OVCAR-3 cells. (**B**) Effects of EGF or TNF on CCL20, CXCL1, CXCL2, CXCL3 and CXCL8 by qRT-PCR in SKOV-3 cells. (**C**) Effects of EGF or TNF on CCL20, CXCL2, CXCL8 and CXCR5 by qRT-PCR in CaOV-3 cells. (**D**) Effects of EGF or TNF on CXCL1, CXCL2, CXCL3 and CXCL8 by qRT-PCR in TOV-21G cells. Cells were incubated with vehicle (Control), EGF (10 ng/ml), TNF (10 ng/ml) or both for 1 h. After isolating total RNA, a qRT-PCR array was performed using specific chemokine primers. An asterisk (*) indicates a significant synergistic effect of the combination of EGF and TNF on chemokine expression (*P* ≤ 0.05) within each group as monitored by ANOVA and Tukey’s pairwise comparisons. Experiments were performed in triplicate and all data are shown as mean ± SEM.

Based on chemokines and chemokine receptors influenced by EGF or TNF in PCR array data (Figures 1, 2, 3 and 4 and Table 1), we confirmed EGF- or TNF-responsive chemokines using qRT-PCR with specific chemokine primers. CCL2, CCL20 and CXCL8 were synergistically elevated (*P* ≤ 0.05) in response to EGF and TNF in OVCAR-3 cells (Figure 5A). On the other hand, CXCL1, CXCL2 and CXCL3 were more responsive to TNF compared to EGF while CXCL16 responded similarly to both EGF and TNF (Figure 5A). Interestingly, although SKOV-3 cells showed a significant synergistic response of CCL20, CXCL1, CXCL2, CXCL3 and CXCL8 (*P* ≤ 0.05) levels to the addition of EGF plus TNF, TNF alone had a greater effect than EGF alone (Figure 5B). Induction levels of CCL20 or CXC8 were larger than those of CXCL1-3 (Figure 5B).

CaOV-3 cells exposed to EGF plus TNF synergistically elevated CXCL8 and CXCR5 (*P* ≤ 0.05), but showed a dominant effect of EGF>TNF, when each was added alone (Figure 5C). CCL20 and CXCL2 levels also underwent a greater increase with EGF added alone, than TNF (Figure 5C). Finally TOV-21G cells induced CXCL1-3 and CXCL8 without any apparent synergistic effect in response to EGF plus TNF. They also showed a greater induction by EGF than TNF (Figure 5D). The synergistic responses observed (Figure 5) were consistent with our PCR array data (Table 1).

### Characteristics of components related with differential EGF- or TNF-activated Akt, Erk and IκB in ovarian cancer cells

**Figure 6:**
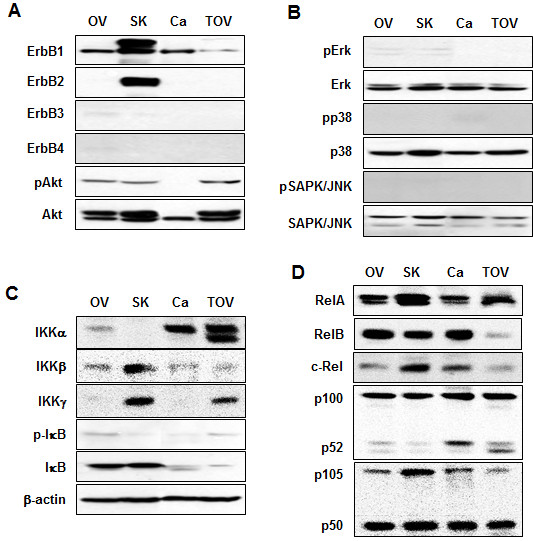
**The baseline expression of signaling components associated with EGF- or TNF-activation.** Expression levels of ErbB isoforms and Akt (**A**), MAPK pathway (**B**), IKK isoforms and IκB (**C**), and NF-κB family (**D**) in OVCAR-3 (OV), SKOV-3 (SK), CaOV3 (Ca) and TOV-21G (TOV) cells. Whole cell lysates were prepared and protein expression levels were confirmed by Western blot analyses. Experiments were performed in duplicate and a representative result is shown.

Based on different responses to EGF or TNF in ovarian cancer cells (Figures 1, 2, 3, 4 and 5), we compared those signaling components previously related with EGF- or TNF-activated Akt, Erk and IκB in ovarian cancer cells. We measured and compared ErbB isoforms, Akt, the MAPK pathway (Erk, p38 and SAPK/JNK), IKK isoforms, IκB, and the NF-κB family in nonstimulated ovarian cancer cells and compared their differential expression patterns. All cell lines expressed ErbB1, a specific receptor for EGF; SKOV-3 also highly expressed ErbB2 (Figure 6A). CaOV-3 cells expressed less Akt (Figure 6A), indicating less EGF-mediated activation of Akt (Figure 3C). Erk, p38 and SAPK/JNK expressions were similar between the cell lines (Figure 6B). Interestingly CaOV3 and TOV-21G cells highly expressed IKKα whereas OVCAR-3 and SKOV-3 cells highly expressed IκB (Figure 6C). In addition, CaOV3 and TOV-21G cells highly expressed p52 as compared to OVCAR-3 and SKOV-3 cells (Figure 6D). These baseline data support our findings that OVCAR-3 and SKOV-3 cells are more responsive to TNF while CaOV3 and TOV-21G cells more responsive to EGF.

### CCL20 and CXCL8 promoter activities in response to EGF and TNF in ovarian cancer cells

**Figure 7:**
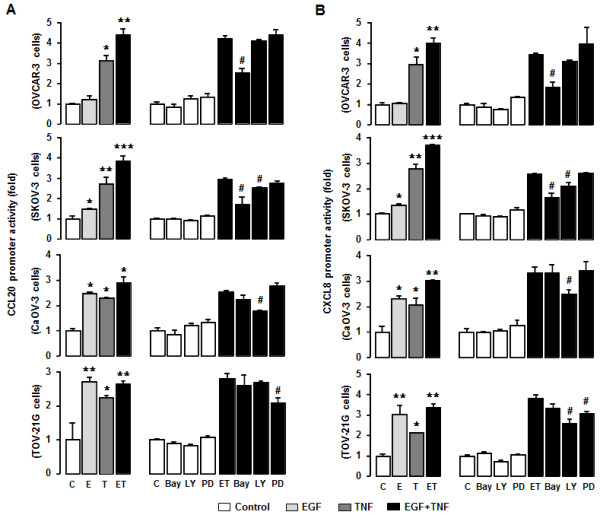
**Promoter activities for EGF- or TNF-responsive CCL20 and CXCL8 in ovarian cancer cells.** CCL20 promoter activity (**A**) and CXCL8 promoter activity (**B**) in response to EGF (E), TNF (T) or both (ET) ± inhibitors for IκB (BAY-11-7082, Bay 2 μM), Akt (LY294002, LY 2 μM) and Erk (PD98059, PD 10 μM) in OVCAR-3, SKOV-3, CaOV3 and TOV-21G cells. After transfection with CCL20 and CXCL8 luciferase vectors overnight, a luciferase assay was performed. Results were normalized to total protein concentrations and expressed as fold increases compared to controls. Different asterisks (*,**,***) indicate significantly increased differences (P ≤ 0.05) within each group by ANOVA and Tukey’s pairwise comparison. A number sign (#) indicates a significant decrease (*P* ≤ 0.05) compared to EGF and TNF (ET) measures in the absence of inhibitors, as calculated by Student’s *t*-test. Experiments were performed in triplicate and all data are shown as mean ± SEM.

The regulation of CCL20, CXCL1-3 and CXCL8 is well known [10]. Our findings indicated that the addition of EGF plus TNF produced a synergistic effect on the levels of CCL20 and CXCL8 in OVCAR-3 and SKOV-3 cells (Figure 5). We therefore generated CCL20 and CXCL8 promoters to determine the effects of EGF and TNF on the regulation of CCL20 and CXCL8 at the promoter level. The data show that CCL20 and CXCL8 promoter activities synergistically responded to EGF plus TNF (*P* ≤ 0.05) in OVCAR-3 cells, although when added separately, TNF had a dominant effect (Figure 7A and 7B). BAY-11-7082 (NF-κB inhibitor) significantly reduced this effect on the CCL20 and CXCL8 promoters in contrast to LY294002 (Akt inhibitor) and PD98059 (Erk inhibitor) (Figure 7 and 7B).

EGF and TNF synergistically induced CXCL8 promoter activity (P ≤ 0.05) and EGF alone had a greater response than TNF alone in CaOV-3 cells (Figure 7B). LY294002 significantly reduced CCL20 and CXCL8 promoter activities whereas BAY-11-7082 and PD98059 had no effect (Figure 7A and 7B). This fact supported that CaOV-3 cells were more sensitive to EGF > TNF, when each was given alone (Figure 5C).

Although CCL20 was only produced at low levels in TOV-21G cells [even in response to EGF and TNF (Figure 4A)], CCL20 promoter activity responded to both EGF > TNF when each was given alone (Figure 7A). BAY-11-7082 and LY294002 had no effect whereas PD98059 significantly reduced CCL20 promoter activity (Figure 7A). While TOV-21G cells increased CXCL8 promoter activity in response to EGF or TNF, this effect was greater in EGF exposed cells (Figure 7B). LY294002 and PD98059 significantly reduced CXCL8 promoter activity whereas BAY-11-7082 had no effect in these cells (Figure 7B). TNF-dominant responsive cells such as OVCAR-3 and SKOV-3 were found to be sensitive to an NF-κB inhibitor while EGF-dominant responsive cells such as CaOV-2 and TOV-21G were sensitive to Akt or Erk inhibitors.

## Discussion

One of the primary findings in this study is that in these four ovarian cancer cell lines, CCL20, CXCL1-3 and CXCL8 were the major chemokines that responded to EGF or TNF by involving NF-κB, Akt and Erk signaling pathways. Particularly, CCL20 and CXCL8 levels were synergistically elevated in response to a combination of EGF plus TNF.

Although the chemokine signature profile was to some degree cell-type specific, the most highly expressed chemokines were as follows: CCL20, CCL28, CXCL1-3, CXCL8 and CXCL16. These experimental findings are supported by clinical data showing that CXCL1 expression levels are greater in ovarian cancer than in normal ovary tissues and are also higher in sera from women with ovarian cancer [18]. CXCL8 has also been shown to be higher in ascites isolated from ovarian cancer patients than in ascites of women with benign tumors [19,20].

We found that CCL28 and CXCL16 were either unresponsive or far less responsive to EGF or TNF. Although CCL28 levels remain unchanged in these ovarian cell lines, TNF has been shown to increase CCL28 in cultured canine keratinocytes [21] and in A549 human airway epithelial-like cells, via NF-κB signaling [22]. These differences indicate that the chemokine network is likely to be differentially regulated in various cell types. In case of CXCL16, only OVCAR-3 cells induced CXCL16 in response to EGF or TNF, although the effect was very small. The effect of TNF on CXCL16 regulation is still controversial in other model systems. TNF increased CXCL16 in human gingival fibroblasts [23] whereas it had no effect in human vascular smooth muscle cells [24] or in human bronchial epithelial cells [25]. CXCL16 contains a functional activator protein-1 (AP-1) binding motif, and PI3K inhibitors and a c-Jun N-terminal kinase (JNK) inhibitor attenuated IL-18-mediated AP-1 binding and CXCL16 promoter-reporter activity [26]. These facts suggest that EGF or TNF may increase CXCL16 via PI3K or JNK activation, respectively, in OVCAR-3 cells.

On the other hand, CCL20, CXCL1-3 and CXCL8 were highly responsive to EGF or TNF. Because these chemokines contain κB sites on their promoters [13], NF-κB signaling is likely to play a primary role as a regulator. Because EGF does not directly activate NF-κB signaling in OVCAR-3 and SKOV-3 cells, a TNF-activated NF-κB pathway appears to be more dominant in these cells compared to EGF mediated pathways. Interestingly, EGFR-activated NF-κB was observed in both CaOV-3 and TOV-21G cells, explaining the dominant responsiveness of EGF in these lines. In addition, these cells had high levels of IKKα and p52 and low levels of IκB, supporting the concept that a non-canonical NF-κB pathway was involved in their low response to TNF. On the other hand, OVCAR-3 and SKOV-3 cells had low levels of IKKα and p52 and high levels of IκB, supporting a canonical NF-κB pathway responsible for their dominant response to TNF.

Actually the relationship between EGFR activation and NF-κB signaling is very controversial in other model systems. For instance, heparin-binding EGF-like growth factor (HB-EGF) inhibited NF-κB activation via PI3K-dependent phosphorylation of Akt in cytokine-stimulated intestinal epithelial cells [27]. In contrast, EGF contributed to NF-κB activity in human proximal tubule cells [28] and in pancreatic cancer [29]. In addition, EGF did not activate NF-κB or alter NF-κB activation by TNF in chondrocytes [30]. Despite either no or little activation of NF-κB, EGF is likely to broadly induce CCL20, CXCL1-3 and CXCL8 through Akt/Erk activation in ovarian cancer cells. In support of this, EGF was found to activate NF-κB and induce CXCL1 in murine squamous cell carcinoma [31]. The fact that an EGF-induced increase in CXCL1 and CXCL8 was decreased by MAPK inhibitors in ovarian cancer cells [16] indicates involvement of Akt or Erk signaling.

In particular, EGF synergistically induced CCL20 and CXCL8 by cooperating with TNF. CXCL8 is well known to be regulated by NF-κB signaling [32]. In addition to NF-κB signaling, TNF-upregulated CXCL8 is likely to involve JNK and the p38 MAPK pathway [33]. EGF has also been found to induce the release of CXCL8 through signaling pathways involving Erk and PI3K in MCF-7 breast carcinoma cells [34]. In addition, EGFR/Erk and AP-1 pathways were found to be involved in MMP12-induced CXCL8 release from the alveolar epithelium [35]. Thus the combination of EGF and TNF in these studies seems to have boosted CXCL8 expression by coordinating NF-κB, Akt and Erk signaling pathways.

Also CCL20 expression is under the direct control of NF-κB in mouse liver [36] and TNF upregulates CCL20 via NF-κB signaling in intestinal epithelial-type cells [37]. The NF-κB site in CCL20 promoter is critical for LDL responsiveness [38]. In addition to NF-κB signaling, the CCAAT/enhancer binding protein β (C/EBPβ) is a critical regulator of CCL20 in normal human keratinocytes [39]. Proinflammatory cytokine IL-6 stimulates CCL20 expression in part through STAT3 activation in primary murine astrocytes [40]. The miR-21 functionally interacts with the 3′UTR of CCL20 mRNA in colorectal cancer cells, resulting in downregulation of CCL20 [41]. IL-17F is able to induce CCL20 via Erk signaling in bronchial epithelial cells [42]. Similarly with TNF, the combination of IL-1 and EGFR ligands synergistically provoked CCL20 in human keratinocytes [43]. Various signaling pathways involved in CCL20 regulation supports the synergistic effects of EGF and TNF on CCL20 expression by collaboratively involving the NF-κB, Akt and Erk signaling pathways.

Thus TNF-dominant responsive cells (OVCAR-3 and SKOV-3) produced a greater synergistic effect on CCL20 and CXCL8 mRNA when compared to EGF-dominant responsive cells (CaOV-2 and TOV-21G cells). This may be due to the fact that CCL20 and CXCL8 promoters contain a κB site as a main responsive element [10]. Furthermore, promoter assays for CCL20 and CXCL8 supported the synergistic effects of EGF and TNF on CCL20 and CXCL8 via collaboration between the NF-κB and Akt/Erk signaling pathways.

Compared to chemokines, it was difficult to find cell-type specific patterns for chemokine receptors in these cell lines. OVCAR-3 and SKOV-3 cells had either no, or a very low expression of chemokine receptors. Also those ovarian cancer cells with a high endogenous expression of TNF, expressed higher levels of CXCR4 than cells with a low TNF expression [11]. Despite a low expression, however, CXCR4 was responsive to EGF or TNF in OVCAR-3 cells. Although a 24 h treatment with EGF enhanced CXCR4 through PI3K/Akt signaling in SKOV-3 cells [17], the basal level of CXCR4 was found to be low in these cells. Further, even though CaOV-3 and TOV-21G cells expressed high levels of CXCR4, EGF or TNF had no effect on CXCR4 expression in these cell lines. The low response to TNF in CaOV-3 and TOV-21G cells is likely due to their poor responsiveness of CXCR4 expression. Because TOV-21G cells highly express CXCL12/CXCR4 and CXCL16/CXCR6 axes in spite of a low response to EGF or TNF, these axes may contribute to cancer progression as described by others [44,45].

**Figure 8:**
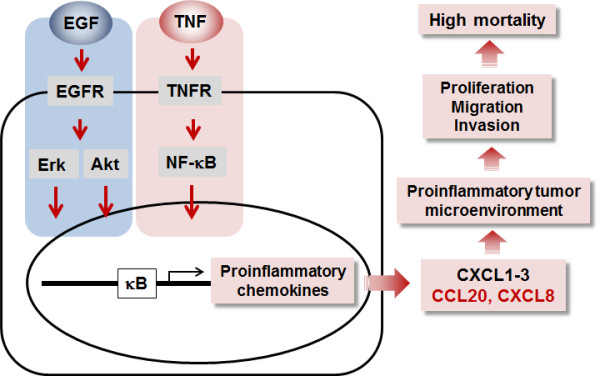
**Schematic proposal of EGF- or TNF-responsive chemokines in ovarian cancer cells.** EGFR, frequently overexpressed in high-grade ovarian cancer, activates Akt and Erk in response to EGF, resulting to secretion of proinflammatory chemokines such as CCL20, CXCL1-3, and CXCL8, all of which contain κB sites in their promoters. Also TNF, a proinflammatory cytokine abundantly expressed in ovarian cancer, induces NF-κB activation followed by secretion of proinflammatory chemokines. Although the response to TNF or EGF is cell-type specific, ovarian cancer cells mainly induce proinflammatory chemokines such as CCL20, CXCL1-3, and CXCL8 through additive or synergistic interaction between EGF and TNF. The enhanced chemokines reinforce the proinflammatory tumor microenvironment for ovarian cancer progression such as peritoneal tumor dissemination and the production of massive ascites which promote the higher mortality rates seen with this malignancy.

Interestingly, CXCR5 was responsive to EGF or TNF in CaOV-3 and TOV-21G cells. Although CXCR5 promotes the growth of tumor cells in the liver [46], the role(s) of CXCR5 in ovarian cancer is poorly understood. As the cancer cells tested do not express CXCL13 (a ligand for CXCR5), the novel role of CXCR5 in ovarian cancer cells in response to EGF or TNF may highlight their interaction with immune cells expressing CXCL13 in the tumor microenvironment. Because inflammation-driven ovarian cancer was found to enhance CXCL1-3 expression [10], - targeting CXCR2, a specific receptor for CXCL1-3, may be a therapeutic strategy as suggested by others [47]. Based on the synergistic increase of CCL20 and CXCL8 in response to EGF and TNF, CCL20 and CXCL8 may be the dominant chemokines found in ovarian cancer cells with abundant TNF, and/or activation of the EGFR. CXCL8 reportedly may function as an important therapeutic target in colorectal cancers [48]. A high expression of CXCL8 was found to be a poor prognostic factor of urothelial bladder cancer [49]. Increased expression levels of CXCL8 may also contribute to the multidrug resistance seen in human breast cancer cells [50]. High expression of CCL20 is also closely associated with poor clinical outcome of patients with gliomas [51] and with a poorer prognosis in patients with hepatocellular carcinoma [52]. The CCL20/CCR6 axis has been shown to promote non-small cell lung cancer progression [53]. Also CCL20 is up-regulated in pancreatic cancer [54] and overexpression of CCL20 in prostate cancer cells promotes tumor growth and invasiveness [55]. Based on these critical roles of CXCL8 and CCL20, high-grade ovarian cancer cells with abundant TNF and EGFR activation may augment these proinflammatory chemokines to provide an inflammatory tumor microenvironment promoting cancer progression and leading to poorer outcomes and an increase in cancer deaths (Figure 8).

## Conclusion

Our results indicate that ovarian cancer cells induce CCL20, CXCL1-3 and CXCL8 as the primary chemokines in response to EGF or TNF. Further, CCL20 and CXCL8 can be significantly elevated by the synergistic actions of EGF plus TNF. Targeting these proinflammatory chemokines may support a promising therapeutic strategy for inflammatory ovarian cancer with abundant TNF and EGFR activation pathways.

## Competing interests

Authors declare no conflict of interest.

## Authors’ contributions

Conceived and designed the experiments: DS and EL. Performed the experiments: DS, SK and YD. Analyzed the data: DS and EL. Contributed reagents/materials/analysis tools: DS, EL and SA. Wrote the paper: DS, EL and SA. All the authors have read and approved the final manuscript.
